# Recovery from Sleeping Sickness

**Published:** 1914-05-02

**Authors:** 


### RECOVERY FROM SLEEPING SICKNESS.

Genuine cases of complete recovery from trypanosomiasis are so extremely rare that much interest attaches to the case-history narrated by Dr. T. S. Kerr in the *Journal of Tropical Medicine and Hygiene*. A lady, who had lived for many years in Portuguese West Africa and had there nursed several cases of sleeping sickness, came to London exhibiting symptoms pointing strongly to a diagnosis of that disease. She had an irregular mottled rash, enlarged spleen, enlarged glands in the neck, axillæ, and groins, deep hyperæsthesia (Kerandel's sign), and felt ill. The provisional diagnosis of trypanosomiasis was confirmed by blood examination. Treatment was at once started; she had soamin injections every fourth day, at first in doses of one grain and increased gradually to three. Sodium antimonium tartrate was taken by the mouth, beginning with half-grain doses, increasing to two grains. Cod-liver oil and open-air exercise in plenty were also ordered. Malaria ensued, and afterwards she began to exhibit intolerance of the antimony, which was accordingly stopped for a time. By this time all the signs and symptoms of trypanosomiasis had disappeared, nor were trypanosomes found in the blood. The weight had steadily increased, and the patient felt well. A year after the treatment began she had occasional attacks of antimonial poisoning, and the dose was then not allowed to exceed half a grain a day. Six months later still an eruption appeared suggestive of sleeping sickness. A quantity of the patient's blood was injected into a monkey, which died with an enlarged spleen wherein trypanosomes were found. After this tartar emetic was injected intravenously; seven doses, ranging from half a grain to a grain and three-quarters, were given. The soamin injections had been given steadily throughout the case. After this, repeated inoculation tests failed to reveal any evidence of trypanosome infection.

